# The Prevalence of Hyperuricemia and Its Correlates among Adults in China: Results from CNHS 2015–2017

**DOI:** 10.3390/nu14194095

**Published:** 2022-10-02

**Authors:** Wei Piao, Liyun Zhao, Yuxiang Yang, Hongyun Fang, Lahong Ju, Shuya Cai, Dongmei Yu

**Affiliations:** Key Laboratory of Trace Element Nutrition of National Health Commission, National Institute for Nutrition and Health, Chinese Center for Disease Control and Prevention, Beijing 100050, China

**Keywords:** hyperuricemia, prevalence, risk factors, adults, China

## Abstract

This study aimed to investigate the prevalence of hyperuricemia (HUA) and associated risk factors in Chinese adults aged 18 to 59 years. All the data were collected from the China Nutrition and Health Surveillance during the period 2015–2017, which adopted a stratified, multistage, random sampling method on a national scale. A total of 52,627 participants aged 18 years or older were included in this study. The definition of hyperuricemia was 420 μmol/L for male and 360 μmol/L for female. The Rao–Scott chi-square test was used to compare the differences in prevalence between or among the subgroups. A weighted two-level multivariate survey-logistic regression was used to detect the correlations between HUA and demographic, physical, lifestyle and metabolic factors. The total prevalence of HUA was 15.1%, and that was higher in male, current smokers, higher BMI and less physical activities subgroups, and also in noninfectious chronic diseases (NCDs) subgroups. The subgroups of non-vegetarian diet, insufficient vegetable intakes and excessive red meat and alcohol intakes had significantly higher HUA prevalence. After introducing all the variables in the survey-logistic regression, gender, age, BMI, physically active, hypertension, diabetes mellitus, bean and nut intake, vegetable intake, red meat intake, alcohol consumption and vegetarian were associated with HUA. Among the significant variables, age and physical active served as a protective factor, and BMI showed to be a risk factor for HUA. Hypertension and dyslipidemia could increase the risk for HUA, while diabetes mellitus was shown a negative association with it. For dietary factors, vegetarian diet, sufficient beans and nuts and vegetables intake could lower the risk of HUA, but more alcohol could increase the risk of HUA. Dietary factor played a key role in HUA. It suggested that the intervention of dietary factor should receive more attention to ameliorate the high prevalence of HUA in China.

## 1. Introduction

As the final product of purine metabolism [1], uric acid (UA) is produced in the liver from degradation of dietary and endogenously synthesized purine compounds. It had been confirmed that the organ systems could be negatively affected by the elevated serum uric acid (SUA) level [2], for the potential effects of UA, such as pro-oxidant, antioxidant, and proinflammatory. However, for the shortage of urate oxidase, UA cannot be degraded to allantoin in the metabolic process of human being. If the producing rate of UA exceed the eliminating rate via kidney, UA could be accumulated and result in high concentration [3]. Hyperuricemia (HUA) is a status of high SUA concentration, which may lead to urate crystals being deposited in joints, tendons, and other tissues, and could also elevate the risk of gout or other comorbidities development [4,5]. Besides gout, the relationships between HUA and dyslipidemia [6], cardiovascular disease (CVD) [7], metabolic syndrome (MetS) [4], insulin resistance [8], hypertension [9] or renal disease [10] have been observed in previous studies.

In China, HUA was also showed that might be a potential risk to public health. In 2015–2016, the total HUA prevalence in China was 11.1% and then elevated to 14.0% in 2018–2019 [11]. For the broad territory and multi-ethnic populations, the prevalence of HUA in China was diverse. For regions, Huang et al. [12] found that the HUA prevalence of urban adults in southwestern China was 13.5%, and it was higher than the HUA prevalence of Henan rural population (12.6%) [13]. The fluctuations in HUA prevalence that have been shown above might suggest that the affect factors for HUA in different regions or populations are quite varied. In previous studies, the associations of HUA with age, gender, socioeconomic conditions, and marital status have been observed [14,15,16]. Additionally, some chronic diseases, for example, hypertension, hyperlipidemia, overweight or obesity were also considered to have the effects on an increased risk for HUA [13,17,18]. For food sources, the purine-rich diet, such as seafoods, meats, and drinking have been considered to be the risk for HUA [19]. Dietary pattens, for instance, the Healthy Cardiometabolic Diets and the Dietary Approaches to Stop Hypertension (DASH) Diet, contributed to the development of HUA [20], and that has also been verified in an animal model [21].

However, while the previous studies have found some of the risk factors for HUA, few of them considered the combinations of demographic information, chronic disease status and diet intaking conditions, and the representativeness of population was also a limitation to them. The current study aims to describe the prevalence of HUA using the data of the China Nutrition and Health Surveillance (CNHS) 2015–2017 [22], a national representative survey, and demonstrate the risk factors for HUA in Chinese adults.

## 2. Materials and Methods

### 2.1. Data Source and Participants

Data of the study were collected from CNHS 2015–2017, which adopted a stratified, multistage, random sampling method to recruit representative participants from 31 provincial-level administrative divisions (PLADs), including provinces, autonomous regions, and municipalities in mainland China [22]. A total of 52,627 participants aged 18 years or older were included in this study.

### 2.2. Data Collection and Measurements

Data were collected from the participants of CNHS 2015–2017 via four parts, including standard questionnaire interview, physical examination, dietary survey, and laboratory test. Questionnaire interview was used to collect the demographic characteristics and lifestyle of participants including the family and personal information by face-to-face interviews. Physical examination was conducted to obtain the height, weight, and blood pressure. Height was measured by the tool of height and sitting height meter (TZG, Suzhou, China), weight was measured by electronic weighing scale (G&G TC-200K, Changshu, China) and blood pressure was measured by Omron HBP-1300 electronic sphygmomanometer (Dalian, China). The consumptions of vegetables, fruits, milk, alcohol, red meat, bean and nut were collected using the method of Food Frequency Questionnaire (FFQ).

Laboratory test was used to detect the levels of serum UA (SUA). Overnight fasting cubital venous blood samples were collected in blood collection tubes (SSTTM II Advance, Becton, Dickinson and Company, Franklin Lakes, NJ, USA) by local CDC staffs and clinicians. After collection, the blood samples were left standing for at least 30 min. Sera were separated from the whole blood by centrifugation at 3000 rpm for 15 min at the survey-site, and then stored at −80 °C until tested in the laboratory. HbA1c (glycosylated hemoglobin α-1-c) was assessed by high-performance liquid chromatography (Premier Hb9210 trinity biotech, Ireland). Fasting blood glucose (FPG), total cholesterol (TC), low-density lipoprotein cholesterol (LDL-C), high-density lipoprotein cholesterol (HDL-C), and triglycerides (TG) were measured by a Hitachi 7600 automatic biochemical analyzer (Hitachi Inc., Tokyo, Japan) with reagents obtained from Wako Pure Chemical Industries Ltd. (Tokyo, Japan) [23] at the central laboratory of the Beijing CDC and National Institute for Nutrition and Health, China CDC. SUA levels were measured using the uricase-HMMPS method on a Roche analyzer (Cobas 8000-c702, Roche Diagnostics, Switzerland).

### 2.3. Quality Control

All implementations of the surveillance were under strict quality control and supervision. Staff of the surveillance should be unified, trained and qualified by the China CDC project team. The face-to-face questionnaire interviews were administered by the qualified investigators, and then questionnaires were checked and verified by inspectors. Physical measurements were operated following the unified manuals or procedures, and the equipment were unified and well calibrated. The blood samples were measured in the designated laboratories using a standard protocol according to the manufacturer’s instructions and reference samples. Data were double input and cleaned up by uniform standard.

### 2.4. Definition of HUA

The definition of HUA was based on the concentration of UA in serum. HUA was diagnosed according to the clinical diagnostic criteria. The cut-off SUA level was 420 μmol/L for male and 360 μmol/L for female [24].

### 2.5. Dietary Intake Assessment

Dietary intake was assessed by 7 kinds of food sources such as bean and nut, vegetable, fruit, milk, red meat, and alcohol. Among these indices, bean and nut, vegetable, fruit, and milk intakes were assessed by the criteria of Chinese Dietary Guidelines (2022): (1) Bean and nut intake was defined as sufficient if the daily average intake was ≥25 g; (2) Vegetable intake was defined as sufficient if the daily average intake was ≥300 g; (3) Fruit intake was defined as sufficient if the daily average intake was ≥200 g; (4) Milk intake was defined as sufficient if the daily average intake was ≥300 g; (5) Red meat intake was categorized as insufficient (daily average red meat intake <18 g), moderate (18 g to 27 g) or excessive (>27 g) [25]. (6) Alcohol consumption was categorized by international guide for monitoring alcohol consumption and related harm (WHO), such as never consumed (consume < 1 g of alcohol per day for both male and female), low risk consumed (consume 1 to 40 g of alcohol per day for male or consume 1 to 20 g of alcohol per day for female), medium risk consumed (consume 41 to 60 g of alcohol per day for male or consume 21 to 40 g of alcohol per day for female) or high and very high risk consumed (consume ≥61 g of alcohol per day for male or consume ≥41 g of alcohol per day for female).

### 2.6. Covariates

All the potential covariates were considered in this study to observe the influent power of dietary factor to HUA. (1) Genders; (2) Residence locations were categorized into urban and rural by region codes (100- for urban and 200- for rural) prescribed by the National Bureau of Statistics; (3) Geographic regions (east, central and west) were defined by National Bureau of Statistics, where east areas included Beijing, Tianjin, Hebei, Liaoning, Shanghai, Jiangsu, Zhejiang, Fujian, Shandong, Guangdong, Hainan, Central areas included Shanxi, Jilin, Heilongjiang, Anhui, Jiangxi, Henan, Hubei, Hunan, and West areas included Inner Mongolia, Guangxi, Chongqing, Sichuan, Guizhou, Yunnan, Tibet, Shaanxi, Gansu, Qinghai, Ningxia, and Xinjiang; (4) Participants were divided into four age groups: 18–29 years, 30–39 years, 40–49 years and 50–64 years; (5) Education level was categorized as low (primary school or below), moderate (junior school) and high (high school or above); (6) According to the annual income of household, four groups were established by conception of middle-level income persons and classification standard in China: low (<65,141.7 CNY), moderate (65,141.7–193,494.9 CNY), high (>193,494.9 CNY) and unknown/unclear; (7) Body mass index (BMI) was categorized as underweight (BMI < 18.5), normal (18.5 ≤ BMI < 24.0), overweight (24.0 ≤ BMI < 28.0), and obese (BMI ≥ 28.0) [26]; (8) Smoking was classed as never smoked, formerly smoked or currently smoke; (9) Physical activity status was decided based on weekly total metabolic equivalent (MET) and total weekly duration of different exercise levels: insufficient (MET < 600), sufficient (600 ≥ MET) [27]; (10) Diabetes mellitus was diagnosed according to the ADA 2010 criteria (FPG level ≥ 7.0 mmol/L and/or HbA1c concentration ≥ 6.5%) [28]; (11) According to the guidelines of Chinese adult dyslipidemia prevention and treatment (2016 revised edition), those with total cholesterol ≥ 6.2 mmol/L or triglyceride ≥ 2.26 mmol/L or LDL ≥ 4.14 mmol/L or HDL < 1.04 mmol/L were considered as dyslipidemia [29]; (12) Hypertensive was identified in those whose mean systolic blood pressure was ≥ 140 mmHg and/or mean diastolic blood pressure ≥ 90 mmHg and/or received antihypertensive medicine within two weeks [30]; (13) Vegetarian was the consumer of plant-based diet with no dairy products and eggs intake.

### 2.7. Statistical Analysis

SAS version 9.4 software (SAS Institute Inc., Cary, NC, USA) was used for all data cleaning and analysis. The demographic structures of subjects were presented as percentages. The weighted prevalence of HUA was presented as ratios and 95% confidence intervals (95% CI), and Rao–Scott chi-square test was used to compare the difference between or among the subgroups. PROC SURVEYLOGISTIC procedure was conducted to detect the influencing factors of HUA, and the results were presented as odds ratio (OR) and 95% CI by comparing with the reference in each model. Model 1 was a crude model, the covariables including gender, residence location, area of the country, age, education level and household income. Model 2 was adjusted for body mass index (BMI), smoking, physical active, hypertension, diabetes mellitus, dyslipidemia. Model 3 was further adjusted for the diet covariables, including bean and nut intake, vegetable intake, fruit intake, milk intake, red meat intake, alcohol consumption and vegetarian. A two-sided *p* value < 0.05 was considered to indicate statistical significance.

### 2.8. Ethics Statements

Written informed consent was obtained from all participants and their guardians. The study protocol was approved by the Ethical Review Committee of the Chinese Center for Disease Control and Prevention (No. 201519-B).

## 3. Results

### 3.1. Basic Characteristics

As shown in Table 1, a total of 52,627 participants (46.41% for males and 53.59% for females) were finally included in current study. Participants who were mid-aged and elderly, had lower household income and normal weight, lived in urban areas and eastern China composed a higher proportion. Compared between different genders, males tended to have higher education level, more physical activities, but more former and current smokers. For those who had hypertension or dyslipidemia, males composed higher proportion, while for diabetes mellitus, females composed higher.

### 3.2. Dietary Intakes

Among whole of the participants, more participants ate a non-vegetarian diet, had less alcohol intakes, but had sufficient intakes of beans and nuts, insufficient intakes of vegetables, fruits and milk, and excessive intakes of red meats. Additionally, except for females tended to have healthier intakes of the above kinds of foods. Further information is available in Table 1.

### 3.3. Prevalence of HUA among Participants

Weighted prevalence of HUA in different categories was shown in Table 2. According to different categories, those who were male, younger, current smokers, and lived in urban areas and eastern China, had higher education level, moderate household income, higher BMI, less physical activities had significantly higher prevalence of HUA. Meanwhile, compared with hypertension or dyslipidemia patients, those who were free of these noninfectious chronic diseases (NCDs) had lower significantly prevalence of HUA. In consideration of food intakes, participants who ate non-vegetarian diet, had insufficient intakes of vegetables, excessive intakes of red meats and alcohol had significantly higher prevalence of HUA.

### 3.4. Influencing Factors of HUA

Results by survey-logistic regression were shown in Table 3. After including all the variables in Model 3, female participants showed less risk of developing HUA (OR = 0.47, 95% CI = 0.41~0.54). Compared with the youngest group, age served as a protective factor (for 30~39 years, OR = 0.63, 95% CI = 0.53~0.76; for 40~49 years, OR = 0.55, 95% CI = 0.46~0.64; for 50~64 years, OR = 0.52, 95% CI = 0.43~0.63). Additionally, being physical active showed a protective factor for HUA (OR = 0.82, 95% CI = 0.73~0.93). Higher BMI showed to be a risk factor for HUA (for overweight, OR = 2.49, 95% CI = 1.52~4.09; for obese, OR = 3.91, 95% CI = 2.38~6.4). For NCDs, hypertension and dyslipidemia could increase the risk for HUA (for hypertension, OR = 1.32, 95% CI = 1.17~1.48; for dyslipidemia, OR = 1.88, 95% CI=1.66~2.12), while diabetes mellitus could decrease it (OR = 0.78, 95% CI = 0.66~0.93). For dietary intake, eating non-vegetarian diet could increase the risk of HUA (OR=1.49, 95% CI=1.15~1.92). Eating sufficient beans and nuts (OR=0.8, 95% CI=0.71~0.9) and vegetables (OR=0.890, 95% CI = 0.797~0.995) could lower the risk of HUA, whereas eating sufficient excessive red meats (OR = 1.36, 95% CI = 1.16~1.59) and more alcohol (for medium, OR = 1.64, 95% CI = 1.09~2.46; for high and very high, OR = 1.39, 95% CI = 1.12~1.73) could increase the risk of HUA. As for residence location, living in rural areas was observed as a protective factor for HUA (OR = 0.78, 95% CI = 0.63~0.96) only in Model 1. Statistical significance was not observed among the other variables.

## 4. Discussion

This study was based on the data from CNHS 2015–2017, and the results were deemed to be national representative. In our study, we observed the HUA prevalence, and further analyzed the influencing factors for HUA. Because of the resulting limitations in previous studies, such as the smaller sample size, region presentiveness or the special population characteristics, the results of this study might be more able to reflect the HUA prevalence status and verify the influencing factors for HUA in Chinese adult population.

The prevalence of HUA was different among populations or regions. In the present study, the overall prevalence of HUA in Chinese adult population was 15.1%, and the prevalence in male (21.2%) was significantly higher than female (8.5%). Data of the National Health and Nutrition Examination Survey (NHANES) showed that the HUA prevalence in the United States in 2015–2016 was 20.1% in total population (20.2% in men and 20.0% in women) [31]. The HUA prevalence in the general Italian population was 11.9% [32]. Additionally, a systematic review demonstrated that the prevalence of HUA in Australia ranged between 10.5% and 16.6% in Caucasian or an Australian representative population [33]. In the neighboring countries, the results drawn from the study that was conducted in the Bangkok population showed that the prevalence of HUA in the study population was 24.4%, and that was significantly more common in male (59%) than female (11%) [34]. The Korean National Health and Nutrition Examination Survey (KNHANES) revealed that the prevalence of HUA in the general Korean population was 11.4% (17.0% in male and 5.9% in female) [35]. From the results shown above, the HUA prevalence in China was lower than that in the US and higher than that in Italy. Moreover, the HUA prevalence of female was nearly equal to male in the US, but that was obviously higher than the Chinese female group. Among the neighboring countries, the prevalence trends of HUA in China were consistent with others. Although the prevalence of HUA in previous domestic studies were fluctuating, the results of our study were consistent with the commonly higher HUA prevalence in male population. In our study, the HUA prevalence in urban and rural was 17.2% and 12.8%, respectively, with both being higher than the results of Yang’s study conducted in Jinan and lower than the results of Zhang’s study conducted in Sichuan province. A study [36] conducted in a China rural population showed that the prevalence of hypertension, dyslipidemia in the HUA group were both higher than that in the non-HUA group. Our results were consistent with these. In our study, the prevalence of HUA was significantly higher in hypertension and dyslipidemia populations, and a negative result was observed in the diabetes mellitus population. The current study showed that the HUA prevalence in the insufficient physical active population was significantly higher than in the sufficient physical active population. However, in Yang’s [30] and Wang’s [37] study, the differences between the insufficient physical active population and sufficient physical active population were not observed. With regard to diet intakes, no positive results of the HUA prevalence were observed in bean and nut intake, fruit intake and milk intake analyses. However, significant differences were detected in vegetable intake, red meat intake and alcohol consumption analyses, being similar to Yang’s and Huang’s studies [12,38]. To our knowledge, our study is the first of its kind to introduce the vegetarian factor, and the prevalence of HUA in the vegetarian group was significantly lower than in the non-vegetarian group.

Although the risk factors for HUA had been detected in previous studies, the effects on HUA were different among them. In the present study, the risk factors for HUA were detected by three survey-logistic regression analysis models. In Model 1, covariates merely included demographic factors. A positive association was observed between the factors of gender, residence location and age and HUA. In Model 2, after adjustment for BMI, smoking, physical active, hypertension, diabetes mellitus and dyslipidemia, the effect of residence location was nullified. Smoking was not observed to have any significant association with HUA. In Model 3, after further adjustment for diet covariables including bean and nut intake, vegetable intake, fruit intake, milk intake, red meat intake, alcohol consumption and vegetarian, the effects of fruit and milk intake were not observed. Among demographic factors, gender and age have been confirmed in nearly all the relevant studies. The underlying biological mechanism could be interpreted as the estrogen concentration fluctuating. Guan’s study [39] showed that in Chinese post-menopausal women population, the HUA prevalence increased remarkably with age increasing. The similar results were also observed in western populations [40,41]. E2 estradiol was inversely associated with the uric acid level [42]. The potential biological mechanisms of E2 affecting the SUA levels may include renal clearance, secretion or reabsorption [43]. That could be the causes of different SUA levels between genders. BMI had been confirmed as a risk factor associated with HUA [44], and that was duplicated in our study. In the results, the prevalence of HUA in obese group was nearly threefold higher than that in the normal group. However, BMI changing may be caused by multiple factors, and more studies should be conducted in further investigations. In the present study, sufficient physical activity was presented as the protective factor for HUA, which was consistent with the results of Cui’s study [17]. The mechanism might be that a lack of exercise may reduce insulin sensitivity and increase urine volume, thus resulting in an elevated SUA level [45]. The associations between hypertension, dyslipidemia and diabetes mellitus and HUA have been detected in previous studies. Yang et al. found that hypertension, hypercholesterolemia and hypertriglyceridemia were the risk factors for HUA [38]. In Zhu’s study, diabetes mellitus was detected as the risk factor for HUA, but that was only observed in the women group. That might be due to the sex-specific physiologic impact of HUA [46]. In our findings, hypertension and dyslipidemia were detected as the risk factor for HUA in our study, which was consistent with previous studies, but the contrary was true for diabetes mellitus. Although the associations between HUA and metabolic diseases have been observed, the potential mechanisms should be further studied.

Diet may play an important role in the development of HUA. The associations between dietary factors and HUA have been detected in several studies. Purine, as one of the most important sources of UA in food components, has been observed in previous studies. A study conducted in The Health Professionals Follow-up Study found that higher consumption levels of purine-rich foods, such as meat and seafood, were associated with an increased risk of gout, and dietary patterns rich in animal foods were positively associated with a higher risk of HUA, whereas the vegetable pattern was negatively associated [47]. Villegas et al. reported that both meat and seafood intake have been associated with higher prevalence of HUA, but no association between total protein intake and HUA was observed [48]. In our study, the higher consumption levels of red meat were positively associated with HUA, and the negative associations between bean and nut intake and vegetable intake and HUA were observed. The vegetarian was also detected as the protective factor for HUA in our results. No associations between fruit and milk intake and HUA were observed in the current study, which was consistent with previous studies. According to a previous study, protein-rich foods might enhance UA production due to the higher quantity of purines. However, this also could promote the urinary urate excretion and lead to a reduction in the concentration of SUA [49]. Higher unsaturated fatty acid and phytochemical-rich plant intakes are the main contents of the principal food sources of the vegetarian diet. By virtue of xanthine oxidase inhibition, phytochemicals have strong effects on reducing SUA levels while inhibiting UA generation in purine metabolism [50]. The relationship between alcohol and HUA was also observed in our study. In comparison with the never consumption group, the OR in the medium consumption group was 1.640 (95 CI%:1.093–2.459) and dropped to 1.390 (95 CI%: 1.117–1.730) in the high- and very high-risk consumption group. This trend was consistent with Choi’s study [51]. One of the potential mechanisms could be the conversion of alcohol to lactic acid, which reduces renal uric acid excretion by competitively inhibiting uric acid secretion by the proximal tubule [52]. However, a mendelian randomization study demonstrated that alcohol consumption had no causal effect on the development of HUA [51]. It suggested that, apart from other factors, genetic backgrounds may potentially influence the HUA prevalence among the different ethnic groups.

As a national representative survey, this study was first time to describe the prevalence of HUA in the Chinese adults population. According to our results, the associations between HUA and gender, age, physically active, hypertension and dietary factors were verified. Because of the population mobility and economic development, the influence strength of geographical, education and household income were vanished. To the best of our knowledge, the present study was the first of its kind to introduce vegetarian as an independent influencing factor into the analysis. However, some limitations of this study should be noticed. Firstly, the present study was cross-sectional research, and thus, the causal relationship between HUA and influence factors may not be well elucidated. Secondly, cancer status, genetic and ethnic factors were not considered in this study, and thus, the effects of cancer and genes on HUA might be ignored.

## 5. Conclusions

HUA is a public health problem in the Chinese adult population, especially in male and urban residents, and it should be paid more attention by the relevant government sectors. Dietary factors, including bean and nut intake, vegetable intake, red meat intake, and alcohol consumption, were significantly associated with HUA. Moreover, vegetarian diet could be a protective factor for HUA. Comparing with other influencing factors of HUA, dietary factors were intervened more feasibly. Besides focusing on a certain type of food item, an optimal dietary pattern could bring about better HUA intervention effects. The outcomes of this study could also provide some notions for the prevention of other chronic diseases and be beneficial for large-scale populations. For the aim of diet conversion, policy making and targeted health education should be taken into account.

## Figures and Tables

**Table 1:** Demographic and clinical characteristics of all participants.

	Male		Female		Overall
	N	%	N	%	N
Total	24,425	46.4	28,202	53.6	52,627
Residence location					
Urban	9576	45.3	11,567	54.7	21,143
Rural	14,849	47.2	16,635	52.8	31,484
Area of the country					
East	9495	46.1	11,117	53.9	20,612
Central	6889	46.3	7979	53.7	14,868
West	8041	46.9	9106	53.1	17,147
Age (years)					
18~29	2493	44.2	3149	55.8	5642
30~39	3534	45.3	4262	54.7	7796
40~49	6595	45.8	7804	54.2	14,399
50~64	11,803	47.6	12,987	52.4	24,790
Education level					
Low	8247	37.0	14,069	63.0	22,316
Moderate	9658	53.7	8326	46.3	17,984
High	6520	52.9	5807	47.1	12,327
Household income					
Low	16,324	46.8	18,593	53.3	34,917
Moderate	3914	46.1	4576	53.9	8490
High	430	46.3	499	53.7	929
Unknown	3757	45.3	4534	54.7	8291
BMI					
Wasting	856	41.4	1211	58.6	2067
Normal	11,133	45.8	13,161	54.2	24,294
Overweight	8907	48.0	9641	52.0	18,548
Obese	3529	45.7	4189	54.3	7718
smoking					
Never	8327	23.3	27,390	76.7	35,717
Former	13,471	95.3	671	4.7	14,142
Current	2627	94.9	141	5.1	2768
Physically active					
Insufficient	12,984	43.3	16,978	56.7	29,962
sufficient	11,441	50.5	11,224	49.5	22,665
Hypertension					
No	15,917	44.5	19,826	55.5	35,743
Yes	8508	50.4	8376	49.6	16,884
Diabetes mellitus					
No	22,381	46.2	26,106	53.8	48,487
Yes	2044	49.4	2096	50.6	4140
Dyslipidemia					
No	13,197	41.6	18,527	58.4	31,724
Yes	11,228	53.7	9675	46.3	20,903
Bean and nut intake					
Insufficient	10,614	44.4	13,305	55.6	23,919
sufficient	13,811	48.1	14,897	51.9	28,708
Vegetable intake					
Insufficient	12,914	45.2	15,645	54.8	28,559
sufficient	11,511	47.8	12,557	52.2	24,068
Fruit intake					
Insufficient	20,441	47.9	22,201	52.1	42,642
sufficient	3984	39.9	6001	60.1	9985
Milk intake					
Insufficient	24,053	46.6	27,604	53.4	51,657
sufficient	372	38.4	598	61.7	970
Red meat intake					
Insufficient	5503	36.6	9523	63.4	15,026
Moderate	1805	40.4	2658	59.6	4463
excessive	17,117	51.7	16,021	48.4	33,138
alcohol consumption					
Never	11,883	31.4	25,927	68.6	37,810
Low risk	8746	81.7	1966	18.4	10,712
Medium risk	1346	89.0	167	11.0	1513
High and very high risk	2450	94.5	142	5.5	2592
Vegetarian					
No	1059	36.9	1808	63.1	2867
Yes	23,366	47.0	26,394	53.0	49,760

**Table 2:** Prevalence rate of HUA in different characteristics subgroups.

	Prevalence % (95% CI)	Rao–Scott X^2^	*p*-Value
Total	15.1 (13.6, 16.6)		
Gender			
Male	21.2 (19.1, 23.4)	696.3878	<0.0001
Female	8.5 (7.5, 9.5)		
Residence location			
Urban	17.2 (14.7, 19.6)	13.9459	0.0002
Rural	12.8 (11.5, 14.0)		
Area of the country			
East	16.9 (14.2, 19.7)	8.3070	0.0157
Central	12.9 (11.2, 14.7)		
West	14.4 (12.5, 16.2)		
Age (years)			
18~29	17.8 (15.2, 20.4)	23.4681	<0.0001
30~39	14.8 (13.2, 16.3)		
40~49	14.0 (11.8, 16.1)		
50~64	13.6 (12.4, 14.7)		
Education level			
Low	11.9 (10.6, 13.3)	74.0552	<0.0001
Moderate	14.8 (13.1, 16.5)		
High	17.9 (15.9, 19.9)		
Household income			
Low	14.3 (12.7, 16.0)	21.6375	<0.0001
Moderate	17.7 (16.0, 19.5)		
High	14.6 (10.6, 18.6)		
BMI			
Wasting	8.0 (5.2, 10.9)	133.0930	<0.0001
Normal	9.9 (7.7, 12.2)		
Overweight	18.2 (16.3, 20.1)		
Obese	27.3 (24.8, 29.8)		
Smoking			
Never	12.5 (11.0, 14.0)	119.7219	<0.0001
Former	20.1 (18.3, 21.8)		
Current	22.9 (18.0, 27.7)		
Physically active			
Insufficient	16.1 (14.3, 17.9)	20.9569	<0.0001
Sufficient	13.2 (11.9, 14.6)		
Hypertension			
No	13.6 (11.9, 15.3)	38.8866	<0.0001
Yes	19.9 (18.0, 21.8)		
Diabetes mellitus			
No	15.0 (13.4, 16.6)	1.2533	0.2629
Yes	16.4 (14.3, 18.5)		
Dyslipidemia			
No	10.2 (8.6, 11.8)	175.3248	<0.0001
Yes	22.9 (21.2, 24.7)		
Bean and nut intake			
Insufficient	15.3 (13.8, 16.9)	0.3804	0.5374
Sufficient	14.9 (13.1, 16.6)		
Vegetable intake			
Insufficient	16.0 (14.1, 17.9)	6.7256	0.0095
Sufficient	14.3 (12.9, 15.7)		
Fruit intake			
Insufficient	15.2 (13.4, 17.0)	0.4620	0.4967
Sufficient	14.6 (13.1, 16.0)		
Milk intake			
Insufficient	15.1 (13.5, 16.6)	0.0624	0.0624
Sufficient	14.5 (10.1, 18.9)		
Red meat intake			
Insufficient	10.8 (9.5, 12.1)	75.4133	<0.0001
Moderate	12.6 (10.1, 15.1)		
Excessive	17.1 (15.4, 18.9)		
Alcohol consumption			
Never	12.2 (11.3, 13.2)	84.3755	<0.0001
Low risk	21.1 (17.0, 25.2)		
Medium risk	25.3 (18.9, 31.8)		
High and very high risk	21.9 (18.8, 25.1)		
Vegetarian			
No	15.4 (13.8, 17.0)	24.3736	<0.0001
Yes	9.1 (7.1, 11.1)		

Abbreviations: BMI, body mass index; CI, confidence interval.

**Table 3:** Associations between risk factors and HUA in the participants.

Influencing Factors	Model 1		Model 2		Model 3	
	OR (95% CI)	*p*-Value	OR (95% CI)	*p*-Value	OR (95% CI)	*p*-Value
Gender		<0.0001		<0.0001		<0.0001
Male	Ref.		Ref.		Ref.	
Female	0.346 (0.311, 0.385)		0.406 (0.346, 0.476)		0.469 (0.406, 0.541)	
Residence location		0.0215		0.0854		0.0706
Urban	Ref.		Ref.		Ref.	
Rural	0.776 (0.626, 0.963)		0.810 (0.637, 1.030)		0.812 (0.648, 1.018)	
Area of the country		0.1097		0.0734		0.1556
East	Ref.		Ref.		Ref.	
Central	0.776 (0.610, 0.986)		0.745 (0.563, 0.987)		0.777 (0.592, 1.021)	
West	0.889 (0.699, 1.130)		0.925 (0.700, 1.221)		0.916 (0.71, 1.183)	
Age (years)		0.0039		<0.0001		<0.0001
18~29	Ref.		Ref.		Ref.	
30~39	0.783 (0.646, 0.948)		0.646 (0.543, 0.769)		0.631 (0.527, 0.756)	
40~49	0.773 (0.666, 0.896)		0.571 (0.485, 0.671)		0.546 (0.463, 0.644)	
50~64	0.749 (0.625, 0.897)		0.532 (0.441, 0.642)		0.518 (0.428, 0.627)	
Education level		0.5866		0.464		0.6253
Low	Ref.		Ref.		Ref.	
Moderate	0.982 (0.870, 1.108)		0.935 (0.827, 1.056)		0.952 (0.841, 1.077)	
High	1.063 (0.882, 1.280)		0.992 (0.823, 1.195)		1.002 (0.832, 1.206)	
Household income		0.1145		0.1334		0.1408
Low	Ref.		Ref.		Ref.	
Moderate	1.116 (0.944, 1.321)		1.138 (0.973, 1.332)		1.114 (0.949, 1.309)	
High	0.821 (0.584, 1.153)		0.897 (0.623, 1.291)		0.849 (0.584, 1.235)	
BMI				<0.0001		<0.0001
Wasting			Ref.		Ref.	
Normal			1.390 (0.796, 2.426)		1.382 (0.815, 2.346)	
Overweight			2.501 (1.500, 4.170)		2.492 (1.519, 4.089)	
Obese			3.879 (2.340, 6.428)		3.906 (2.384, 6.398)	
smoking				0.2249		0.3176
Never			Ref.		Ref.	
Former			1.036 (0.877, 1.223)		0.939 (0.751, 1.173)	
Current			1.210 (0.968, 1.512)		1.142 (0.934, 1.397)	
Physically active				0.0036		0.0014
Insufficient			Ref.		Ref.	
sufficient			0.844 (0.754, 0.946)		0.824 (0.732, 0.927)	
Hypertension				<0.0001		<0.0001
No						
Yes			1.314 (1.171, 1.476)		1.317 (1.172, 1.480)	
Diabetes mellitus				0.0099		0.0056
No			Ref.		Ref.	
Yes			0.789 (0.659, 0.945)		0.783 (0.659, 0.930)	
Dyslipidemia				<0.0001		<0.0001
No			Ref.		Ref.	
Yes			1.853 (1.634, 2.102)		1.878 (1.664, 2.120)	
Bean and nut intake						0.0002
Insufficient					Ref.	
sufficient					0.801 (0.713, 0.899)	
Vegetable intake						0.0404
Insufficient					Ref.	
sufficient					0.89 (0.797, 0.995)	
Fruit intake						0.1793
Insufficient					Ref.	
sufficient					0.881 (0.732, 1.060)	
Milk intake						0.3563
Insufficient					Ref.	
sufficient					0.823 (0.545, 1.245)	
Red meat intake						0.0001
Insufficient					Ref.	
Moderate					1.106 (0.861, 1.420)	
excessive					1.356 (1.160, 1.585)	
alcohol consumption						0.0106
Never					Ref.	
Low risk					1.278 (0.917, 1.781)	
Medium risk					1.640 (1.093, 2.459)	
High and very high risk					1.390 (1.117, 1.730)	
Vegetarian						0.0023
No					Ref.	
Yes					0.672 (0.520, 0.867)	

Abbreviations: BMI, body mass index; OR, odds ratio; CI, confidence interval.

## Data Availability

The data presented in this study are non-public.
